# Impact of antimicrobial stewardship programs on antibiotic consumption and antimicrobial resistance in four Colombian healthcare institutions

**DOI:** 10.1186/s12879-022-07410-6

**Published:** 2022-05-02

**Authors:** Christian Pallares, Cristhian Hernández-Gómez, Tobías Manuel Appel, Kevin Escandón, Sergio Reyes, Soraya Salcedo, Lorena Matta, Ernesto Martínez, Sara Cobo, Laura Mora, Adriana Marín, Adriana Correa, Elsa De La Cadena, Jesús Rodríguez-Baño, María Virginia Villegas

**Affiliations:** 1 Grupo de Investigaciones en Resistencia Antimicrobiana y Epidemiología Hospitalaria (RAEH), Universidad el Bosque, Bogotá, Colombia; 2 Centro Internacional de Entrenamiento e Investigaciones Médicas (CIDEIM), Cali, Colombia; 3 Clínica Imbanaco Grupo Quirónsalud Cali, Cali, Colombia; 4 Division of Infectious Diseases and International Medicine, University of Minnesota Medical School, Minneapolis, MN USA; 5 Clínica General del Norte, Barranquilla, Colombia; 6 Corporación Clínica Universitaria Comfenalco, Cali, Colombia; 7 Universidad del Valle, Cali, Colombia; 8 DIME Clínica Neurocardiovascular, Cali, Colombia; 9 Universidad Santiago de Cali, Cali, Colombia; 10 Unidad Clínica de Enfermedades Infecciosas, Microbiología y Medicina Preventiva, Hospital Universitario Virgen Macarena, Seville, Spain; 11 Departamento de Medicina, Universidad de Sevilla, Seville, Spain

**Keywords:** Antimicrobial stewardship, Antimicrobial stewardship program, Antimicrobial resistance, Antibiotic resistance, Hospital epidemiology, Colombia

## Abstract

**Background:**

Antimicrobial stewardship programs (ASPs) have become a fundamental pillar in optimizing antimicrobial usage, improving patient care, and reducing antimicrobial resistance (AMR). Herein we evaluated the impact of an ASP on antimicrobial consumption and AMR in Colombia.

**Methods:**

We designed a retrospective observational study and measured trends in antibiotic consumption and AMR before and after the implementation of an ASP using interrupted time series analysis over a 4-year period (24 months before and 24 months after ASP implementation).

**Results:**

ASPs were implemented according to the available resources in each of the institutions. Before ASP implementation, there was a trend toward an increase in the antibiotic consumption of all measured antimicrobials selected. Afterward, an overall decrease in antibiotic consumption was observed. The use of ertapenem and meropenem decreased in hospital wards, while a decrease in the use of ceftriaxone, cefepime, piperacillin/tazobactam, meropenem, and vancomycin was observed in intensive care units. After ASP implementation, the trend toward an increase of oxacillin-resistant *Staphylococcus aureus*, ceftriaxone-resistant *Escherichia coli*, and meropenem-resistant *Pseudomonas aeruginosa* was reversed.

**Conclusions:**

In our study, we showed that ASPs are a key strategy in tackling the emerging threat of AMR and have a positive impact on antibiotic consumption and resistance.

## Background

Antimicrobial resistance (AMR) is considered a global threat to public health and [1, 2] and is responsible for more than 700,000 deaths per year. By 2050, it could reach figures of up to 10 million deaths annually [3] and could harm the gross domestic product of the countries, especially low- and middle-income countries (LMICs) [4].

The high adaptability of microorganisms and the relation between antimicrobial misuse and AMR have been known for many decades [5]. In 1996, McGowan and Gerding called for “antimicrobial-use stewardship” consisting in optimizing antimicrobial selection and dosage and duration of treatment in order to respond to the emerging threat of AMR [6]. Over the last years, antimicrobial stewardship programs (ASPs) have become a fundamental pillar in optimizing antimicrobial usage by improving compliance to antimicrobial guidelines and are known to improve patient care, while having a favorable impact on AMR [7, 8].

As LMICs usually have higher rates of AMR due to a lack of rapid diagnostic tests, last-generation antimicrobials, and epidemiological surveillance [9], ASP-oriented strategies such as online training, mentoring programs, national guidelines, and use of social media platforms have become a priority [8]. However, the consolidation of these ASPs is challenging due to the frequent shortage of healthcare professionals trained in antimicrobial stewardship, lack of electronic medical records, as well as the absence of national public health policies to confront AMR [9].

Several hospital-based studies focusing on inpatients have demonstrated that ASPs can increase adherence to antimicrobial therapy guidelines and reduce consumption of unnecessary antibiotics, while having a favorable impact on AMR rates, hospital-acquired infections, and patient outcomes [8, 10–12]. The most effective interventions include prospective audit and feedback, preauthorization, and facility-specific treatment recommendations [13]. Although successful experiences of ASPs have been published in Latin America, there are only few reports on the clinical, microbiological, and economic impact of these interventions [14–18].

The objective of the present study was to evaluate the impact of ASPs on antibiotic consumption and AMR in four high-complexity hospitals in Colombia using interrupted time series analysis.

## Methods

A retrospective observational study was conducted in four high-complexity hospitals (Institutions A–D) in two Colombian cities (Cali and Barranquilla) over 48 months from 2009 to 2012 (24 months before and 24 months after ASP implementation). Antibiotic consumption and the incidence of meropenem-resistant *Acinetobacter baumannii* (MEM-R *Aba*), ceftriaxone-resistant *E. coli* (CRO-R *Eco*), ertapenem-resistant *K. pneumoniae* (ETP-R *Kpn*), meropenem-resistant *P. aeruginosa* (MEM-R *Pae*), and oxacillin-resistant *Staphylococcus aureus* (OXA-R *Sau*) was measured during the study period. A baseline ASP evaluation was performed at the beginning of the study period, followed by an evaluation over the next six months to monitor the ASP progress using the *indicateur composite de bon usage des antibiotiques* (ICATB) antimicrobial stewardship index [19]. An average ICATB score was calculated. General wards and intensive care units (ICUs) were included in the analysis. Emergency rooms and pediatric wards were excluded from the study.

Common features of the ASPs in the participating institutions included: (1) A multidisciplinary ASP team: infectious disease physicians, pharmacists, microbiologists, head nurses, and the infection control and prevention committee; (2) Antimicrobial guidelines for the most prevalent infections, which were updated by the ASP team and were based on the institution’s epidemiology; (3) A consensus of the antimicrobial guidelines was reached among the different specialists after discussion and before implementation; (4) Prospective audit and feedback was the strategy in all but one institution (Institution D implemented a restrictive prescription control model); (5) Prescriptions for the chosen audited antibiotics was reviewed by the ASP team (mainly by a general practitioner who reported to the infectious disease physician) once antibiotic therapy was started, with direct feedback and recommendations to continue, adjust, change, or discontinue the therapy; (6) Educational interventions were carried out periodically (every 4–6 months) to remind physicians of the antimicrobial guidelines; and (7) Hospital administration supported the ASM team interventions.

Defined Daily Doses (DDDs) based on the World Health Organization (WHO) calculation system were used to measure antibiotic consumption (https://www.whocc.no/atc_ddd_index/). The DDDs per 100 occupied bed-days were recorded pre- and post-intervention for ceftriaxone, cefepime, piperacillin/tazobactam, ertapenem, meropenem, and vancomycin in each hospital monthly. A global measure for all hospitals was generated every month during the evaluation period.

To measure the incidence of MEM-R *Aba*, CRO-R *Eco*, ETP-R *Kpn*, MEM-R *Pae*, and OXA-R *Sau*, the number of patients with hospital-acquired infections (according to the US Centers for Disease Control and Prevention [CDC] surveillance system criteria) who had a positive microbiological culture was divided by the number of admissions per hospital (in a 6-month period) × 1000 patient admissions. Only one isolate of the same species per patient was included. On the other hand, no major changes in hand hygiene, isolation precautions, and cleaning and disinfection strategies were recorded in the four hospitals. Protocols implemented by the infection control and prevention committees remained identical during the time evaluated.

The 2009 and 2010 Clinical & Laboratory Standards Institute (CLSI) guidelines were used to determine the resistance trends, considering the susceptibility breakpoints at the time of the study for each isolate, thereby guaranteeing the comparability of the results.

Interrupted time series analysis was used to compare the global monthly DDD antibiotic use and the cumulative incidence over six months of MEM-R *Aba*, CRO-R *Eco*, ETP-R *Kpn*, MEM-R *Pae*, and OXA-R *Sau* in hospital wards and ICUs. Antibiotic consumption, coefficient and incidence of infections pre-intervention, trends in pre- and post-intervention, and changes in the absolute post-intervention level were recorded. The following definitions were used: *β*_*0*_ as the constant, *β*_*1*_ as the coefficient of the pre-intervention trend, *β*_*2*_ as the change in the trend, and *β*_*3*_ as the post-intervention trend [20]. Statistical analyses were conducted in STATA^®^ version 15. p values < 0.05 were considered statistically significant.

## Results

### Characteristics of ASPs in the four institutions

Four hospitals were included during 48 months of follow-up; their characteristics are shown in Table 1.

**Table 1:** Characteristics of the four institutions that participated in this study

	Institutions			
	A	B	C	D
City	Barranquilla	Cali	Cali	Cali
Number of hospital beds	500	450	900	150
Ownership	Private	Public	Public	Private
Clinical services	General hospital*	General hospital*	General hospital*	Cardiovascular and neurosurgery
Organ transplantation	No	No	Bone marrow	No

*All general hospitals had emergency departments, general wards, intensive care units, and general and specialized surgery

ASPs had a different human resources distribution across hospitals, although all programs were led by either an epidemiologist or an infectious disease physician (Table 2). The average cost of the ASP was 1143 USD per 100 patient-beds. Institution D and B dedicated the longest time to the ASP interventions with 122.93 and 120.67 working hours per month per 100 patient-beds, respectively. Consistently, the number of working hours dedicated by infectious disease physicians, epidemiologists, and hospital pharmacists was higher in these two institutions. The ASP in institution D, with a monthly average of 2158 USD per 100 patient-beds, was the most expensive program among the 4 institutions due to more dedication by specialists.

**Table 2:** Characteristics of the ASP of the four participating institutions

Resources	Institutions			
	A	B	C	D
Total working hours dedicated to the ASP per month per 100 beds	35	120.67	41.67	122.93
Working hours dedicated to the ASP per month per 100 beds by				
Infectious disease physicians	3	4.44	2.22	26.67
Epidemiologists	0	28.44	0.44	42.67
General practitioners	0	42.67	29.33	26.67
Nursing staff	24	12.89	8	0.27
Hospital pharmacist	1.6	12.89	1	26.67
Microbiologists	2.4	6.44	0.67	0
Administrative assistants	4	12.89	0	0
Average monthly cost per 100 beds (in USD)	493.8	1312.67	609.78	2158
Average ICATB score	17.5	17.75	19.75	16.65

### Antibiotic consumption and bacterial resistance

**Fig. 1:**
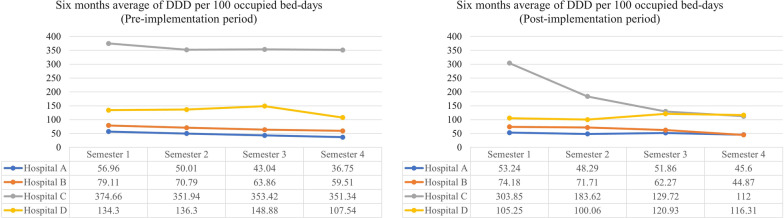
Trend of DDD per 100 occupied bed-days) for consumption of broad-spectrum antibiotics (ceftriaxone, cefepime, piperacillin/tazobactam, ertapenem, meropenem, and vancomycin) in ICUs and general wards

Prior to the implementation of the ASPs, the four institutions showed a trend towards an increase in broad-spectrum antibiotic consumption (ceftriaxone, cefepime, piperacillin/tazobactam, ertapenem, meropenem, and vancomycin) in general wards and ICUs (Fig. 1). After the ASP implementation, all institutions decreased antibiotic consumption; the greatest reduction was achieved in institution B (45%), followed by institutions A (29%), D (28%), and C (20%). Institution C reversed the trend in antibiotic consumption, at levels even lower than the first studied period in comparison with the third semester of the post-implementation period (p < 0.001). After the implementation of the ASP, institutions C, D, and B had a significant decrease to 49%, 16%, and 7% for meropenem, cefepime, and ceftriaxone consumption (p < 0.001), respectively. No statistically significant difference was observed in the consumption of vancomycin, piperacillin/tazobactam, and ertapenem. In the case of institution A, a decrease in the consumption of meropenem, piperacillin/tazobactam, and ceftriaxone was observed in the first year post-ASP implementation although the behavior did not show any decreasing trend in the following year (p > 0.05).

Before the implementation of the ASPs in hospital wards, a statistically significant upward trend was observed in all the antibiotics evaluated. After the ASP implementation, there was a statistically significant decrease in the consumption of ertapenem and meropenem. However, no statistically significant decrease was observed in the consumption of the other antibiotics (Table 3). Regarding the ICUs, before the implementation of the ASPs, a statistically significant upward trend was observed in all the antibiotics evaluated, except ertapenem and vancomycin. After the ASP implementation, a decrease was found for ceftriaxone, cefepime, piperacillin/tazobactam, meropenem, and vancomycin.

**Table 3:** Antibiotic consumption (in DDD per 100 occupied bed days) in general wards and intensive care units pre- and post-intervention

	Value	General wards		Intensive care units	
		Coefficient (95% CI)	*p*	Coefficient (95% CI)	*p*
CRO	*β*_*0*_	14.44 (12.87 to 16)	< 0.001	22.7 (20.32 to 25.09)	< 0.001
	*β*_*1*_	0.39 (0.22 to 0.55)	< 0.001	0.23 (0.06 to 0.4)	0.01
	*β*_*2*_	− 2.51 (− 6.05 to 1.03)	0.16	− 11.07 (− 14.02 to − 8.12)	< 0.001
	*β*_*3*_	− 0.69 (− 0.93 to − 0.43)	< 0.001	− 0.39 (− 0.6 to − 0.17)	0.001
FEP	*β*_*0*_	18.33 (14.46 to 22.2)	< 0.001	104.06 (88.59 to 119.54)	< 0.001
	*β*_*1*_	0.96 (0.55 to 1.37)	< 0.001	1.96 (0.97 to 2.94)	< 0.001
	*β*_*2*_	− 5.45 (− 17.42 to 6.51)	0.36	− 73.17 (− 89.77 to − 56.56)	< 0.001
	*β*_*3*_	− 1.17 (− 1.99 to − 0.35)	0.006	− 2.06 (− 3.98 to − 1.23)	0.02
TZP	*β*_*0*_	7.36 (5.51 to 9.21)	< 0.001	31.97 (27.13 to 36.81)	< 0.001
	*β*_*1*_	0.56 (0.37 to 0.75)	< 0.001	0.93 (0.56 to 1.3)	< 0.001
	*β*_*2*_	− 3.19 (− 7.64 to 0.21)	0.16	− 26.83 (− 38.59 to − 15.08)	< 0.001
	*β*_*3*_	− 0.49 (− 0.77 to − 0.21)	0.001	− 0.93 (− 1.69 to − 0.18)	0.02
ETP	*β*_*0*_	11.67 (8.86 to 14.48)	< 0.001	19.06 (13.84 to 24.29)	< 0.001
	*β*_*1*_	0.52 (0.35 to 0.7)	< 0.001	− 0.33 (− 0.61 to − 0.05)	0.02
	*β*_*2*_	− 6.25 (− 9.51 to − 2.99)	< 0.001	1.67 (− 2.05 to 5.4)	0.37
	*β*_*3*_	− 0.54 (− 0.75 to − 0.32)	< 0.001	0.28 (− 0.02 to 0.57)	0.07
MEM	*β*_*0*_	11.89 (8.77 to 15)	< 0.001	179.82 (162 to 197.65)	< 0.001
	*β*_*1*_	1.2 (0.9 to 1.5)	< 0.001	1.8 (0.79 to 2.82)	0.001
	*β*_*2*_	− 15.92 (− 23.28 to − 8.57)	< 0.001	− 129.23 (− 144.8 to − 113.66)	< 0.001
	*β*_*3*_	− 0.75 (− 1.27 to − 0.23)	0.006	− 1.55 (− 3.01 to − 0.09)	0.04
VAN	*β*_*0*_	6.24 (4.55 to 7.93)	< 0.001	107.73 (95.77 to 119.68)	< 0.001
	*β*_*1*_	0.41 (0.27 to 0.55)	< 0.001	0.44 (− 0.47 to 1.35)	0.34
	*β*_*2*_	− 2.91 (− 6.14 to 0.33)	0.08	− 65.78 (− 80.56 to − 51)	< 0.001
	*β*_*3*_	− 0.53 (− 0.82 to − 0.25)	< 0.001	− 1.09 (− 2.06 to − 0.13)	0.03

*β*_*0*_ baseline consumption, *β*_*1*_ baseline trend, *β*_*2*_ change in consumption after intervention, *β*_*3*_ change in trend after intervention, *CI* confidence interval, *CRO* ceftriaxone, *ETP* ertapenem, *FEP* cefepime, *MEM* meropenem; *TZP* piperacillin/tazobactam, *VAN* vancomycin

As for multidrug-resistant bacteria, prior to the implementation of the ASPs, there was a statistically significant upward trend for OXA-R *Sau*, MEM-R *Pae*, and CRO-R *Eco*. In contrast, the trends for ETP-R *Kpn* and MEM-R *Aba* were not statistically significant. After the implementation of the ASPs, the trend changed for CRO-R *Eco*, MEM-R *Pae*, and OXA-R *Sau*, while the trend was not statistically significant for MEM-R *Aba* and ETP-R *Kpn* (Table 4).

**Table 4:** Incidence of MEM-R *Aba*, CRO-R *Eco*, ETP-R *Kpn*, MEM-R *Pae*, and OXA-R *Sau* per 1000 patient admissions in Colombian healthcare institutions pre- and post-intervention

Value	MEM-R *Aba*		MEM-R *Pae*		CRO-R *Eco*		ETP-R *Kpn*		OXA-R *Sau*	
	Coefficient (95% CI)	*p*	Coefficient (95% CI)	*p*	Coefficient (95% CI)	*p*	Coefficient (95% CI)	*p*	Coefficient (95% CI)	*p*
*β*_*0*_	6.45 (3.34 to 9.55)	0.004	3.39 (3.16 to 3.62)	< 0.001	3 (2.97 to 3.03)	< 0.001	0.51 (− 0.13 to 1.14)	0.09	5.61 (4.96 to 6.28)	< 0.001
*β*_*1*_	− 0.91 (− 2.67 to 0.86)	0.23	0.33 (0.18 to 0.48)	0.004	0.18 (0.15 to 0.21)	< 0.001	0.17 (− 0.18 to 0.52)	0.25	0.35 (0.06 to 0.64)	0.03
*β*_*2*_	− 3.24 (− 10.12 to 3.64)	0.26	− 0.67 (− 1.28 to − 0.06)	0.04	− 1.16 (− 1.44 to − 0.88)	< 0.001	0.17 (− 1.07 to 1.41)	0.72	− 4.64 (− 5.6 to − 3.86)	< 0.001
*β*_*3*_	1.3 (− 0.37 to 2.97)	0.10	− 0.62 (− 0.82 to − 0.43)	< 0.001	− 0.67 (− 0.77 to − 0.58)	< 0.001	0.05 (− 0.5 to 0.59)	0.82	− 0.55 (− 0.84 to − 0.26)	0.006

*β*_*0*_ baseline incidence, *β*_*1*_ baseline trend, *β*_*2*_ change in incidence after intervention, *β*_*3*_ change in trend after intervention, *CI* confidence interval, *CRO-R Eco* ceftriaxone-resistant *E. coli*, *ETP-R* *Kpn* ertapenem-resistant *K. pneumoniae*, *MEM-R* *Aba* meropenem-resistant *A. baumannii*, *MEM-R Pae* meropenem-resistant *P. aeruginosa*, *OXA-R* *Sau* oxacillin-resistant *S. aureus*

## Discussion

The implementation of ASPs and the optimal usage of antibiotics are critical to curb AMR [8, 21]. In our study, we observed a reduction in the consumption of selected antimicrobials in three of the four institutions studied. Several strategies implemented by the hospitals may have contributed to the success of the ASPs in these hospitals. The fact that the ASPs were constituted by an interdisciplinary team of professionals was critical since these were in charge of socializing, implementing, and measuring the adherence to the antimicrobial guidelines. Additional successful strategies included discussing the antimicrobial guidelines with the prescribing specialists before implementing the ASP and introducing instruments for monitoring antibiotic consumption, which helped follow closely any changes in antimicrobial prescription.

The healthcare institutions that implemented ASPs had to adapt their interventions to the available human resources and salary support for the antimicrobial stewardship team. Our experience was similar to what Perozziello and colleagues reported in French hospitals [22]. Another key element was the support of the hospital administration in the study institutions, facilitating the governance of the ASP working team. Also, assigning working hours to specialists in infectious diseases, hospital pharmacists, general practitioners, and nursing staff is an essential element for the successful implementation of the ASPs [23]. In institutions B and C, the high percentage of working hours dedicated by general practitioners to the implementation of the ASP may have contributed to high adherence to their antimicrobial guidelines, similar to what Goff and colleagues have reported [24]. In institution C, a head nurse was in charge of monitoring the adherence and administration of antimicrobials, providing daily feedback to the physicians. The excellent results obtained in the ASP run by nurses when there are few or only one infectious disease specialist for 800 beds are similar to the findings published by Monsees [25].

After the implementation of the ASPs in the general wards of the four healthcare institutions in Colombia, a decreasing trend in the consumption of all antibiotics studied was observed but it only achieved statistical significance for carbapenems. The use of carbapenems has been previously associated with collateral damage, selecting for multidrug-resistant bacteria [26–29]. Therefore, reducing its consumption will have an impact on the incidence of resistant microbiota in the hospital as well as cost savings.

In this study, the implementation of ASPs showed a decrease in the incidence of CRO-R *Eco*, OXA-R *Sau*, MEM-R *Pae*, and MEM-R *Aba*. Other studies in Colombia have also shown a decrease in extended-spectrum β-lactamase (ESBL)-producing *E. coli*, as well as an increase in resistance to third-generation cephalosporins [15, 16]. Studies have also reported a decrease in the incidence of MEM-R *Pae* after the implementation of an ASP [16, 18], as well as for other antibiotics such as piperacillin/tazobactam and cefepime [15, 16]. The design of this study cannot prove that the results in bacterial resistance are exclusively attributable to the implementation of ASP. Other factors influencing the decrease in resistant bacteria may include increases in adherence to hand hygiene and cleaning and disinfection practices, as well as general awareness of AMR which may or may not be related to the implementation of this study.

The value of hospital ASPs may be very different across countries. However, in a systematic review Dilip et al. [30] showed that the mean cost savings varied by hospital size and region after implementation of ASPs. Average cost savings in US studies were 732 USD per patient (range 2.50–2640), with similar trends in European studies. In our study, the monthly average of the most expensive program was 2158 USD per 100 patient-beds due to the time dedicated by healthcare professionals, with 122.93 working hours/month per 100 patient-beds.

We realize that studies of ASP interventions have several limitations. Measuring variables such as favorable clinical outcomes or long-term decrease in bacterial resistance are difficult to link with the ASP strategies used partly due to the relatively short time of measurement since the implementation of each ASP. On the other hand, changes over the years in the local epidemiology of AMR may influence the results of any study. Furthermore, statistical analysis cannot capture the effects that occurred before the ASP interventions [31].

Nevertheless, in our study, we used interrupted time series analysis in which the level and trend of the pre-intervention segment serve as the control for the post-intervention segment, providing a methodologically acceptable design for measuring the effect of an intervention. As the interruption in the time series refers to the specific point at which the intervention was implemented, the inference that an intervention directly affects the outcome in the post-intervention period is strengthened by the presence of a control group that has never had the intervention and therefore shows no changes from the pre-intervention to post-intervention period. In addition, the time series design may control for time-dependent confounding effects such as seasonality [32, 33]. The evaluation of ASPs with interrupted time series analysis is increasingly necessary due to the need for standardization of strategies, outcome indicators, and a standardized way of measurement, as well as the fact that time models can be more robust when evaluating ASPs. Despite all the advantages of this methodology, there are some limitations. The number of observations, the symmetry of the data before and after the intervention, and a high autocorrelation of the data can affect the power of the study. Therefore, if a statistically significant decrease in antibiotic consumption and a decrease in bacterial resistance are reported over time, the statistical model does not allow us to know which of the multiple strategies implemented during the ASP was the most effective since all the ASP strategies were simultaneously implemented.

## Conclusions

Antimicrobial stewardship is critical to tackling the emerging threat of AMR. Evaluation of ASPs is increasingly reported in the literature but methodological flaws in the design, analysis, and reporting of these interventions hinder the interpretation and wider implementation of apparently successful interventions. Although the number of large-scale ASPs is rapidly growing internationally, LMICs struggle to demonstrate the success of such programs. High-quality interrupted time series analysis studies may help analyze ASP interventions despite some intrinsic limitations. In our study that compared four hospital ASPs, we were able to show that it is possible to implement such programs in LMIC hospital settings. We further demonstrated that ASPs played a key role in decreasing antibiotic consumption and drug resistance. We consider that ASPs must have nationwide regulatory support as a public health policy, bearing in mind that they are also currently part of the measurable elements of accreditation for hospitals related to patient safety.

## Data Availability

All findings from the data analyzed during this study are included in this published article. Data that support our findings are available from the corresponding author upon reasonable request.

## Abbreviations

AMR: Antimicrobial resistance
ASP: Antimicrobial stewardship program
CDC: US Centers for Disease Control and Prevention
CLSI: Clinical & Laboratory Standards Institute
CRO-R *Eco*: Ceftriaxone-resistant *Escherichia coli*
DDD: Defined daily dose
ESBL: Extended-spectrum β-lactamase
ETP-R *Kpn*: Ertapenem-resistant *Klebsiella pneumoniae*
ICATB: *Indicateur composite de bon usage des antibiotiques*
ICU: Intensive care unit
LMIC: Low- and middle-income country
MEM-R *Aba*: Meropenem-resistant *Acinetobacter baumannii*
MEM-R *Pae*: Meropenem-resistant *Pseudomonas aeruginosa*
OXA-R *Sau*: Oxacillin-resistant *Staphylococcus aureus*
WHO: World Health Organization
